# In vivo Diagnosis of Primary Cutaneous Amyloidosis —The Role of Reflectance Confocal Microscopy

**DOI:** 10.3390/diagnostics9030066

**Published:** 2019-06-27

**Authors:** Ana-Maria Ionescu, Mihaela-Adriana Ilie, Virginia Chitu, Andrei Razvan, Daniela Lixandru, Cristiana Tanase, Daniel Boda, Constantin Caruntu, Sabina Zurac

**Affiliations:** 1 First Department of Dermatology, Colentina Clinical Hospital, 020125 Bucharest, Romania; 2 Dermatology Research Laboratory, “Carol Davila” University of Medicine and Pharmacy, 050474 Bucharest, Romania; 3 Department of Biochemistry, “Carol Davila” University of Medicine and Pharmacy, 050474 Bucharest, Romania; 4 Department of Dermatology, “Carol Davila” University of Medicine and Pharmacy, 050474 Bucharest, Romania; 5 Department of Pathology, Colentina Clinical Hospital, 020125 Bucharest, Romania; 6 Department of Biochemistry-Proteomics, “Victor Babes” National Institute of Pathology, 050096 Bucharest, Romania; 7 Department of Dermatology, “Prof. N. Paulescu” National Institute of Diabetes, Nutrition and Metabolic Diseases, 011233 Bucharest, Romania; 8 Department of Physiology, “Carol Davila” University of Medicine and Pharmacy, 050474 Bucharest, Romania

**Keywords:** cutaneous amyloidosis, in vivo, non-invasive, reflectance confocal microscopy

## Abstract

Primary cutaneous amyloidosis (PCA) is a form of localized amyloidosis. It is characterized by the deposition of a fibrillar material in the superficial dermis, without affecting other systems or organs. The diagnosis can be made clinically, but usually a skin biopsy is performed in order to exclude other skin diseases with similar appearance. Reflectance confocal microscopy (RCM) is a novel imaging tool that enables in vivo characterization of various skin changes with a high, quasi-microscopic resolution. This technique might have an important role in the differential diagnosis of cutaneous amyloidosis, by the in vivo assessment of epidermal changes and dermal amyloid deposition. Moreover, it is completely non-invasive and can be safely repeated on the same skin area. However, to date, there is only one published paper presenting the confocal features of primary cutaneous amyloidosis. Hereby, we describe the in vivo RCM features of PCA lesions from a patient with diabetes and correlate them with histologic findings. This strengthens the clinical usefulness of in vivo RCM examination for the non-invasive diagnosis of cutaneous amyloidosis, especially in patients that might associate diseases with impaired wound healing.

## 1. Introduction

Primary cutaneous amyloidosis (PCA) represents a form of localized amyloidosis, characterized by the deposition of a fibrillar β-sheet folding structure material in the superficial dermis. The involvement of other internal organs is absent due to the local synthetization and deposition of the proteic aggregates; therefore, it does not imply a vital risk [1,2,3]. However, the impact on the quality of life can be significant, whereas intense pruritus is frequently associated and the skin appearance might be perceived as unpleasant [3,4]. Clinical features of PCA define the three subtypes of the disease: macular, papular (lichenoid form), and nodular amyloidosis [1,2,5]. Clinical appearance varies among the three forms of PCA: macular PCA is characterized by hyperpigmented areas of the skin, which can be confluent or have a wavy pattern, associated with pruritus; papular PCA is presented as intense pruritic papules that have the tendency to coalesce. These two forms may coexist, defining the biphasic amyloidosis. The nodular form consists of infiltrated nodules or plaques. The localization of the lesions usually follows extensor surface of the extremities and the trunk [1,6].

PCA is usually clinically diagnosed, especially in regions with a high prevalence, such as Asia and Middle East. However, in cases with non-specific clinical appearance or in less common affected regions, as Europe, a skin biopsy is required for diagnosis [3,6,7].

The pathological examination of macular and papular PCA shows eosinophilic material in the papillary dermis, with a green birefringence under polarized light after Congo red staining [1,8,9,10]. At this level, melanophages, pigment incontinentia, and scattered perivascular lymphohistiocytic infiltrate may be present [1,5]. The suprajacent epidermis may present acanthosis and hyperkeratosis [8]. In nodular form, the amyloid deposition can be present in the profound dermis and subcutis or even affecting blood vessel walls [8]. Other diagnostic tools available are keratin immunohistochemical staining for macular and papular PCA [1,2,5] and immunohistochemical staining for immunoglobulin light chains in nodular PCA [1].

Reflectance confocal microscopy (RCM) is a non-invasive imaging tool that enables in vivo epidermal and superficial dermal characterization, with a high, quasi-microscopic resolution [11]. The images are created at variable depths, in horizontal slices—en face. The acquired images are in a gray-scale coloring and they are obtained live, allowing a quick examination. Also, the tissue of interest does not require any preparation, so the scan can be easily and rapidly done [12]. This imaging technique has been extensively used for the non-invasive diagnosis of melanocytic [13,14] and non-melanocytic lesions [12,15,16,17,18,19] and also for the evaluation of various inflammatory dermatologic conditions [20,21,22,23,24] or cutaneous inflammatory processes in experimental settings [25,26].

In PCA, RCM investigation may play an important role for the non-invasive diagnosis of cutaneous amyloidosis, by enabling the visualization of the dermal deposition of amyloid and the epidermal changes [6]. In addition, RCM may be a useful tool for the evaluation of the treatment response, by allowing repeated investigations of the same skin area [11].

## 2. Case Report

We present the case of a 62-year-old man who was admitted to the “Prof. Dr. Nicolae C. Paulescu” National Institute of Diabetes, Nutrition and Metabolic Diseases, Bucharest, Romania and was referred to the Department of Dermatology for the assessment of a pruritic eruption on his lower legs. Written informed consent was provided and the patient agreed to undergo the diagnostic and therapeutic procedures included in the study protocol that was conducted in accordance with the Declaration of Helsinki with approval No.25/27.11.2017 of Colentina Clinical Hospital Research Ethics Committee.

**Figure 1:**
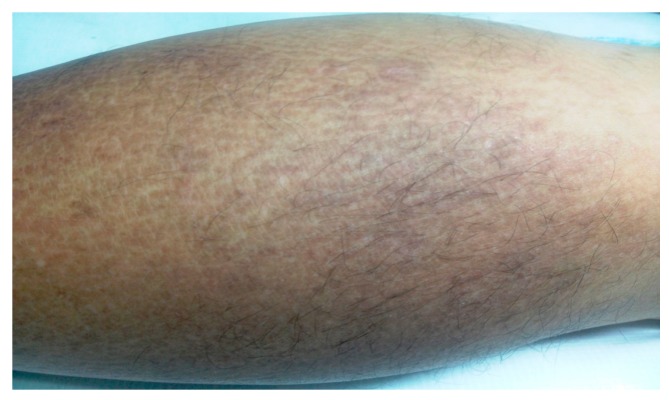
Clinical image showing brownish macules with a rippled pattern on the lower leg.

The patient’s medical history consisted of uncontrolled type 2 diabetes mellitus, diagnosed 2 years previously and benign prostate hypertrophy. The skin lesions occurred approximately 2 years before the presentation and were initially interpreted as scabies and treated accordingly. The pruritus did not improve and the cutaneous modifications progressively became more evident. The physical examination revealed a Fitzpatrick IV skin phototype, brownish macules with a rippled pattern coalescing and forming poorly delineated hyperpigmented areas on the calves (Figure 1). Differential diagnosis based on clinical examination included post-inflammatory hyperpigmentation, atrophic lichen planus, and drug-induced pigmentation. Systemic amyloidosis was excluded by detailed investigation for additional amyloid deposits.

In vivo RCM was performed in the affected area, using the commercially available reflectance confocal microscope Vivascope 1500 (Lucid-Tech Inc., Henrietta, NY, USA), with a wavelength of 785 nm. This system uses a laser beam with near-infrared wavelengths and less than 30 mW power to provide horizontal, gray-scale images of the skin at different levels, down to a depth of 200–250 µm. A single field of view, usually of 500 × 500 µm, has a lateral resolution of approximately 1 µm and an axial resolution of 3–5 µm [27]. With this device, images are obtained with a rate of 9 frames per second and enables real-time micromorphologic and dynamic imaging of skin structures [16,28]. Acquisition method implies the use of a magnetic tissue ring that is attached to the head of the microscope on one side and to the skin surface by means of an adhesive plastic window on the other side. These provide tissue stabilization and prevent transmission of pressure in the investigated skin area. Crodamol oil or water is placed between the skin and plastic window and transparent ultrasound gel between the plastic window and the microscope head [29].

**Figure 2:**
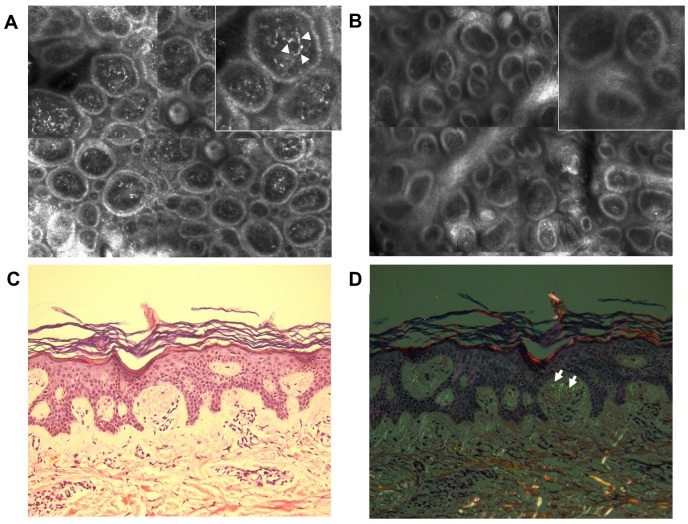
Correlation between reflectance confocal microscopy (RCM) and histological images: (**A**) RCM mosaic (1.5 × 1 mm) and detail (0.25 × 0.25 mm) from the dermoepidermal junction level of the affected skin showing highly refractile structures with various shapes (dotted, coliform, and agglomerates) inside dermal papillae (white short arrows). (**B**) RCM mosaic (1.5 × 1 mm) and detail (0.25 × 0.25 mm) from the dermoepidermal junction level of clinically unaffected skin. Histopathological images showing (**C**) purple amyloid deposits in the papillary dermis (×20, Congo red stain) and (**D**) the green-yellow birefringence (white short arrows) of amyloid deposits inside dermal papillae (×20, Congo red stain, polarized microscopy).

**Figure 3:**
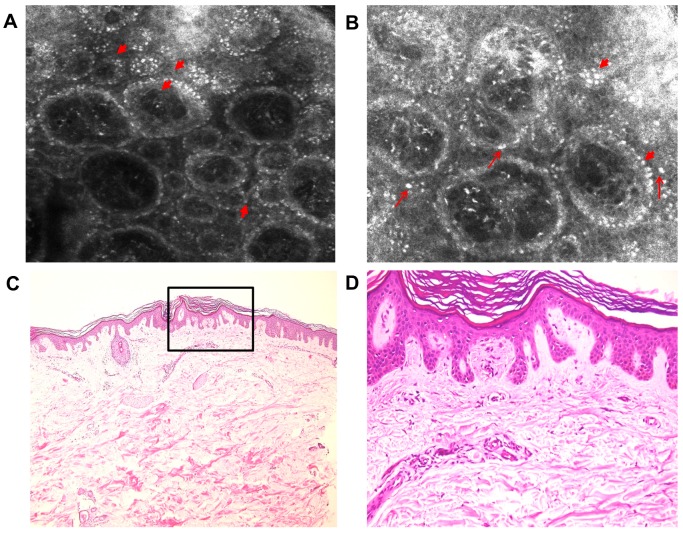
Correlation between RCM and histological images. RCM images at the dermal–epidermal junction level (**A**) (0.5 × 0.5 mm) and (**B**) (0.4 × 0.4 mm) showing highly refractile basal cells due to increased melanin (short red arrows) and bright plump, oval to stellate-shaped cells (thin red arrows). Histopathological images (**C**) (HE, ×4) and (**D**) (HE, ×20) showing increased melanin deposition in the basal layer and scant inflammatory reaction in the superficial dermis.

We acquired five mosaics of 5 × 5 mm at different depths (with the Vivablock function of the reflectance confocal microscope) and in the areas of particular interest, full-resolution individual images were collected. The pictures were examined by an experienced dermatologist. RCM identified highly refractile structures inside dermal papillae and in the superficial dermis, with various shapes: dotted, coliform, agglomerates, etc. (Figure 2A). In addition, in the basal layer, increased melanin was present (Figure 3A,B). The epidermis was slightly acanthotic with associated hyperkeratosis. A skin biopsy (4-mm punch biopsy) of the same area previously evaluated with RCM was carried out.

The histological examination of the skin specimens in standard hematoxylin–eosin staining enabled the identification of increased melanin in the basal layer (Figure 3C,D) and slight acanthosis and hyperkeratosis in the epidermis, as well as the presence of amorphous eosinophilic material in the superficial dermis, corresponding to amyloid deposits. A Congo red stain was performed and showed amyloid deposits in a purple hue (Figure 2C). The dyed specimen was analyzed under polarized microscopy, which revealed a slight green birefringence of amyloid deposits (Figure 2D).

The pathological characteristics of the skin sample along with the cutaneous manifestations established the diagnosis of macular amyloidosis. The RCM examination enabled the identification of features, which were also highlighted by the histopathologic analysis, including highly refractile structures with different shapes inside dermal papillae and superficial dermis, corresponding to amyloid deposits. In addition, it enhanced other cutaneous characteristics of amyloidosis, such as increased melanin in the basal layer of epidermis and acanthosis and hyperkeratosis of the epidermis.

## 3. Discussion

Primary cutaneous amyloidosis, like the case presented here, can have an important impact on the patient’s functionality, due to the local symptomatology and the skin appearance. Therefore, a quick and accurate diagnosis has an important and obvious role in the management of patients. In this case, RCM has been demonstrated to be very useful in the patient’s diagnosis, because of the non-invasive character of the examination. The non-invasiveness is even more valuable in patients with impaired wound healing. As in the above-mentioned case, uncontrolled diabetes mellitus slows down the healing process and it increases the risk of biopsy site infections, especially when lower limb is the area of interest. Therefore, by using RCM, not only is the discomfort and possible complication produced by the skin incision avoided, but it also allows the examination of the same affected area countless times, which would be of great use in monitoring the treatment response as well.

Another advantage of RCM in assessing PCA is represented by its dynamic morphological evaluation. The resolution of the gained images is almost comparable with optical microscopy, having the possibility of in vivo visualization of the cells and tissues, without any prior cutaneous preparation [12]. Moreover, the evaluated area can be of a higher dimension than the classical biopsy specimen, by creating live mosaics of the acquired images, up to 8 × 8 mm^2^ [30].

The RCM examination in this case showed increased melanin in the basal layer of epidermis and acanthosis and hyperkeratosis of the epidermis. Also, the highly refractile structures with different shapes in the superficial dermis, corresponding to amyloid deposits, established the diagnosis of PCA without differentiating between clinical subtypes.

The diagnosis was confirmed by a histopathological exam, which showed amorphous material in the dermis, positive for purple hue in Congo red stain and green birefringence under polarized microscope, features specific for the amyloid deposition [1]. Thus, RCM could be an alternative to skin biopsy and pathologic examination, because the magnification is large enough to permit the study of the tissue at a cellular level, in a horizontal plane [11]. Another important aspect of RCM is the real-time manner of evaluation, which may promote a faster diagnosis, followed by a rapid onset of the appropriate treatment [6].

The only published study to date regarding RCM examination in PCA was conducted on 20 Chinese patients with macular and papular amyloidosis [6]. All RCM examinations performed showed high refractive index agglomerates, with dotted, coliform, roped, or cloud-shaped form. Melanin was present in the basal layer, with acanthotic and hyperkeratotic suprajacent epidermis—results similar to the case we presented in this paper. The patients in the mentioned study underwent skin biopsies, all of which confirmed the PCA diagnosis [6].

However, there are some shortcomings when determining the specificity and sensitivity of the technique and its use in PCA cases with atypical appearance. Most of them are related to the small number of the patients enrolled in the studies, due to the fact that RCM is not widely available and the prevalence of cutaneous amyloidosis in some regions, such as Europe, is low. Otherwise, the procedure is safe, repeatable, quick, and non-invasive, and the results in PCA, the macular and papular forms, are reproducible and similar to those available in literature. Hence, in addition to clinical exam, RCM would be a great diagnostic tool in order to avoid skin biopsy. Furthermore, RCM in the evaluation of the therapeutic response may be very useful, but additional research is required.

Another disadvantage of RCM in PCA is represented by the impossibility of differentiating between the clinical forms of the disease, especially those with atypical presentations [6]. The maximum depth of the RCM examination is the superficial dermis, so this could be a limitation in the evaluation of nodular PCA. In order to standardize this procedure for PCA evaluation, further studies are needed, especially for atypical clinical presentations.

## 4. Conclusions

Despite the fact that PCA is not a life-threating disease, it can affect the quality of life of the patients. Therefore, a quick and accurate diagnosis may improve the disease management. RCM, in correlation with the clinical manifestations, represents a great tool in establishing the diagnosis without any further skin biopsy. The RCM evaluation is easily accepted by the patient and it highlights the amyloid deposition in dermis and the subsequent epidermal modification, as in the macular PCA case we presented. RCM is of great use in diabetic patients, especially for those with uncontrolled disease, where skin biopsy may pose a supplementary risk for infection and wound healing impairment. There are some limitations of RCM use in PCA diagnosis; thus, further research is encouraged.
